# Comparative genomics of *Pseudomonas fluorescens* subclade III strains from human lungs

**DOI:** 10.1186/s12864-015-2261-2

**Published:** 2015-12-07

**Authors:** Brittan S. Scales, John R. Erb-Downward, Ian M. Huffnagle, John J. LiPuma, Gary B. Huffnagle

**Affiliations:** Division of Pulmonary and Critical Care Medicine, Department of Internal Medicine, University of Michigan Medical School, Ann Arbor, MI USA; Department of Microbiology and Immunology, University of Michigan Medical School, Ann Arbor, MI USA; Department of Pediatrics and Communicable Diseases, University of Michigan Medical School, Ann Arbor, MI USA

**Keywords:** *Pseudomonas fluorescens*, Genomics, Human, Lung, Metal, Efflux pumps, Cystic fibrosis

## Abstract

**Background:**

While the taxonomy and genomics of environmental strains from the *P. fluorescens* species-complex has been reported, little is known about *P. fluorescens* strains from clinical samples. In this report, we provide the first genomic analysis of *P. fluorescens* strains in which human vs. environmental isolates are compared.

**Results:**

Seven *P. fluorescens* strains were isolated from respiratory samples from cystic fibrosis (CF) patients. The clinical strains could grow at a higher temperature (>34 °C) than has been reported for environmental strains. Draft genomes were generated for all of the clinical strains, and multi-locus sequence analysis placed them within subclade III of the *P. fluorescens* species-complex. All strains encoded type- II, −III, −IV, and -VI secretion systems, as well as the widespread colonization island (WCI). This is the first description of a WCI in *P. fluorescens* strains. All strains also encoded a complete I2/PfiT locus and showed evidence of horizontal gene transfer. The clinical strains were found to differ from the environmental strains in the number of genes involved in metal resistance, which may be a possible adaptation to chronic antibiotic exposure in the CF lung.

**Conclusions:**

This is the largest comparative genomics analysis of *P. fluorescens* subclade III strains to date and includes the first clinical isolates. At a global level, the clinical *P. fluorescens* subclade III strains were largely indistinguishable from environmental *P. fluorescens* subclade III strains, supporting the idea that identifying strains as ‘environmental’ vs ‘clinical’ is not a phenotypic trait. Rather, strains within *P. fluorescens* subclade III will colonize and persist in any niche that provides the requirements necessary for growth.

**Electronic supplementary material:**

The online version of this article (doi:10.1186/s12864-015-2261-2) contains supplementary material, which is available to authorized users.

## Background

Members of the *P. fluorescens* species-complex have versatile metabolic capabilities, allowing them to thrive in a large range of environments [1–4]. Strains of *P. fluorescens* produce a wide-range of secondary metabolites that are important for their association with plants, such as phenazine, polyketides, cyclic lipopeptides, biosurfactants, phytohormones and metabolites that alter plant hormone levels. However, many traits of *P. fluorescens* also benefit its survival in a mammalian host, such as the production of siderophores and bioactive metabolites and the ability to form biofilms. While environmental strains of *P. fluorescens* have optimal growth temperatures between 4 °C and 28 °C, there are documented cases of *P. fluorescens* strains with an expanded growth temperature range that allow them to colonize humans [5–8] or interact with human cells *in vitro* [9–11].

An increasing amount of evidence suggests that some strains of *P. fluorescens* are members of the human microbiota and interact with the human host in ways that contribute to certain chronic diseases (reviewed in [1]). For example, there is a strong link between the presence of circulating antibodies to the *P. fluorescens-*specific peptide I2 and such autoimmune diseases as Crohn’s disease, celiac disease, chronic granulomatous disease and ankylosing spondylitis. We have previously reported that in the absence of acute disease, *P. fluorescens* is cultured from clinical respiratory specimens at a low rate in a hospital setting and is common in asymptomatic lung transplant recipients [12]. *P. fluorescens* strains are often isolated from respiratory specimens from individuals with cystic fibrosis (CF), though this is often unreported (unpublished observation). Additional culture-independent analyses have identified *P. fluorescens* as a low-abundance member of the human microbiome.

There is a large degree of genetic diversity among bacterial strains classified as *P. fluorescens*, which likely reflects the wide range of growth capabilities in these bacteria. Using multi-locus sequence analysis (MLSA) and 16S rRNA gene classification approaches, the *P. fluorescens-*species complex can be divided into three smaller taxonomic subclades [1–4, 13, 14]. Loper et al.*,* used ten housekeeping genes (*acsA, aroE, dnaE, guaA, gyrB, mutL, ppsA, pyrC, recA,* and *rpoB*) to divide seven fully sequenced strains within the *P. fluorescens*-species complex into these three distinct subclades [3]. Subclade I includes *P. chlororaphis* 30*–*84, *P. chlororaphis* O6 and *P. protegens* Pf-5; subclade II includes *P. fluorescens* Pf0-1, *P. fluorescens* Q2-87 and *P. brassicacearum* Q8r1-96; subclade III includes *P. fluorescens* A506, *P. fluorescens* SBW25, and *Pseudomonas sp.* (now known to be *P. synxantha*) BG33R. As expected, strains within a subclade share a higher proportion of conserved domains than the species-complex as a whole. While the core genome of the *P. fluorescens* species-complex was found to be comprised of 2,789 genes, the core genomes of subclades I, II and III were found to be comprised of 4,188, 3,729 and 3,893 genes, respectively [3]. Comparative genomic analysis revealed that these subclade divisions correlate with potentially important functional differences. For example, the genes necessary to produce a type-III secretion system (T3SS) were found in subclades II and III, but not subclade I [3].

Much work has been done on the taxonomy and genomics of the *P. fluorescens* species-complex, but as of yet, no strain isolated from a mammalian source has been sequenced. Here we present the first comparative genomic analysis of *P. fluorescens* strains isolated from humans. The seven *P. fluorescens* clinical strains were isolated from respiratory specimens from individuals with cystic fibrosis (CF), and represent the first publically available draft genomes of human-associated *P. fluorescens* strains. Phylogenetic analysis reveals that these seven clinical strains fall within subclade III of the *P. fluorescens* species-complex. Our analysis reveals an extremely high degree of similarity between the clinical and environmental isolates; however, there are a number of genomic differences between the clinical and environmental subclade III strains, likely reflecting selective pressures unique in the CF lung.

## Results and discussion

### Collection of *P. fluorescens* isolates

The clinical strains of *P. fluorescens* described in this analysis were isolated over a seven-year period from respiratory samples collected at five different hospitals across the United States between March 2001 and January 2008. Six were isolated from adults with CF (five from sputum samples and one from a throat swab). One was isolated from an infant with CF (Table 1). Initial sequence-based analysis of the 16S rRNA-encoding gene at the time of isolation was consistent with *P. fluorescens* and indicated that they were not *P. aeruginosa* (data not shown). Since each isolate came from different CF patients from five different hospitals across a seven-year period, each isolation likely represents a distinct bacterial strain.

**Table 1:** Date, location and source of *P. fluorescens* strains

Isolate ID	Isolation Date	Isolation Location	Isolation Source
AU2989	4/4/01	Hartford, CT	CF Throat Swab, Adult
AU6026	7/28/03	Seattle, WA	CF Sputum, Adult
AU10973	4/6/06	Salt Lake City, UT	CF Sputum, Adult
AU11518	7/12/06	Hartford, CT	CF Sputum, Infant
AU14440	10/19/07	Little Rock, AR	CF Sputum, Adult
AU14705	11/13/07	Augusta, GA	CF Sputum, Adult
AU14917	1/11/08	Little Rock, AR	CF Sputum, Adult

Date, location and source of clinical *P. fluorescens* subclade III strains. The newly sequenced strains were isolated over a seven year period, between March 2001 and Janurary 2008. Isolation occurred at five different hospitals across the United States. Each strain was isolated from a separate patient with CF

### Phenotypic and growth properties

The phenotypic and growth properties of the newly sequenced clinical strains were compared to those of a previously sequenced representative *P. fluorescens* strain, SBW25. The clinical strains all displayed phenotypic characteristics of bacteria within the *P. fluorescens*-species complex, e.g., motile, gram-negative, coccobacillus, lactose-fermentation negative, citrate growth positive, and fluorescence under ultra violet (UV) light (data not shown). While the clinical strains were phenotypically similar to the environmental strains in the above listed traits, they differed in their ability to grow at temperatures above 30 °C. Environmental strains of *P. fluorescens* typically have an optimal growth range between 22 and 28 °C [1]. After 24 h, all of the clinical strains were able to grow at 34 °C or higher in the laboratory, while the environmental subclade III strain SBW25 was unable to grow at this elevated temperature (data not shown). Other investigators have reported that human isolates of *P. fluorescens* have an increased temperature range for growth [1, 5–8], consistent with our observations. The newly sequenced clinical strains are able to grow at temperatures at or above 32 °C but otherwise show the same phenotypic and growth characteristics of previously studied strains of *P. fluorescens*.

### Genomic assembly

The seven clinical *P. fluorescens* strains were sequenced on the Illumina HiSeq platform. Genomic DNA was prepared for each strain and the paired-end read sequences were partially *de novo* assembled using DNAstar’s SeqMan NGen software. Assembly statistics of each of the newly sequenced strains are shown in Additional file 1: Table S1. Contigs were assembled into scaffolds. Average coverage across genome ranged from 44X (AU10973) to 68X (AU14705). As a methodologic control (sequencing and assembly), we also sequenced and *de novo* assembled SBW25, which was included in our subsequent analyses.

### Genomic features

A summary of the genomic features of the *P. fluorescens* strains is shown in Table 2. Genome size of the clinical strains ranged between 6.1 megabase pairs (Mbps) and 6.88 Mbps. This is similar to the range seen in environmental strains (5.95 - 6.72 Mbps). The GC content per genome was also similar between clinical and environmental strains (59.5 - 60.8 % versus 59.6 - 60.5 %, respectively). All of the 16S rRNA gene sequences in the clinical strains were >97 % nucleotide identical to that found in *P. fluorescens* strain A506, with some being >99 % nucleotide identical (Additional file 2: Table S2).

**Table 2:** Genomic features of the *P. fluorescens* strains in this study

	Genome Size (Mbp)	G+C Content (%)	# of Contigs & Scaffolds	# of Coding Sequences	# of RNAs	Plasmid
AU2989	6.2	60.4	49	5569	58	No
AU6026	6.11	60	34	5427	67	No
AU10973	6.13	60.8	23	5499	61	No
AU11518	6.31	60.3	61	5684	63	No
AU14440	6.88	59.5	109	6123	71	No
AU14705	6.1	59.9	97	5379	69	No
AU14917	6.82	60.1	36	5592	68	No
A506 [3]	5.96	60	2	5267	69 (tRNAs)	Yes (57.0)
SBW25 [3]	6.72	60.5	2	5921	66 (tRNAs)	No
BG33R [3]	6.29	59.6	4	5511	68 (tRNAs)	No
SS101 [3]	6.17	60	2	5374	68 (tRNAs)	No

### Phylogenetic analysis

**Fig. 1:**
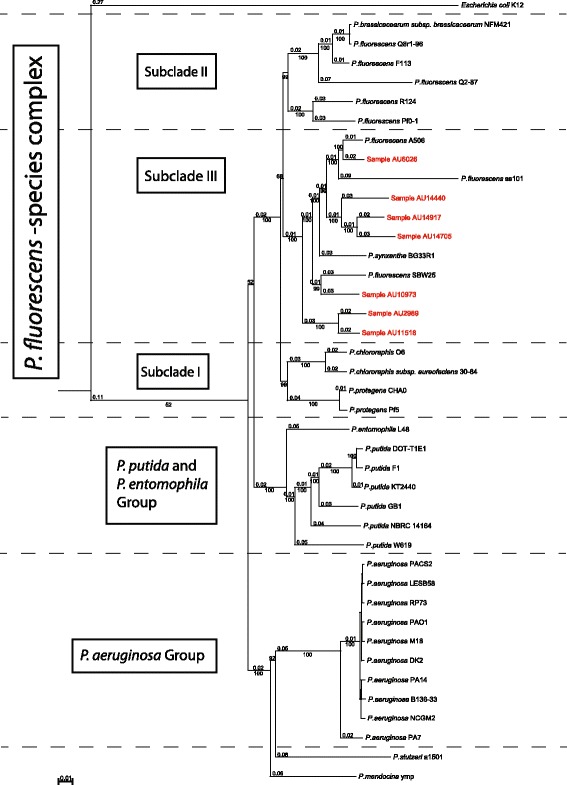
Phylogenetic tree of the *P. fluorescens* strains in this study. Multi-locus sequence analysis of the following eight housekeeping genes was used to infer the phylogenetic tree: *dnaE* (DNA polymerase III alpha subuit); *ppsA* (phosphoenolpyruvate synthase); *recA* (Recombinase A); *rpoB* (RNA polymerase subunit beta); *gyrB* (DNA gyrase subunit B); *guaA* (GMP synthease); *mutL* (DNA mismatch repair protein); *pyrC* (pyrimidine biosynthetic enzyme dihydroorotase) and *acsA* (acetyl-CoA synthetase). The concatenated sequences were aligned with MAFFT as described in the materials and methods. Clinical strains are highlighted in red [80, 81]

To classify the newly sequenced clinical strains, we utilized a multi-locus sequence analysis (MLSA) approach, modified from the scheme described by Loper et al. [3] (Fig. 1). All publically available genomes from the *P. fluorescens* species-complex, and some additional strains within the *Pseudomonas* genus, were included in the phylogenetic analysis. The diverse environmental sources of the fully sequenced *P. fluorescens* isolates have been summarized in a recent review [1]. In our analysis, the previously sequenced strains of *P. fluorescens* formed three distinct phylogenetic subclades within the tree, consistent with the initial publication of the phylogenetic analysis of these strains (Fig. 1) [3, 15].

All seven clinical strains were placed within subclade III of the *P. fluorescens* species - complex (Fig. 1). Strain AU6026, isolated from CF sputum in Seattle, WA (Table 1), segregated closest to environmental strain A506, which was taken from a leaf surface isolate in California [3]. Clinical strain AU10973, isolated from a patient in Salt Lake, UT in 2006, segregated closest with strain SBW25, isolated from a sugar beet in England. Two separate sub-branches within subclade III contained only clinical strains. AU14440, AU14917 and AU14705 branched together on one sub-branch while AU2989 and AU11518 branched together on a second, clinical-only sub-branch. Figure 1 presents the phylogenetic analysis of sequenced clinical strains of *P. fluorescens* alongside sequenced environmental strains.

**Fig. 2:**
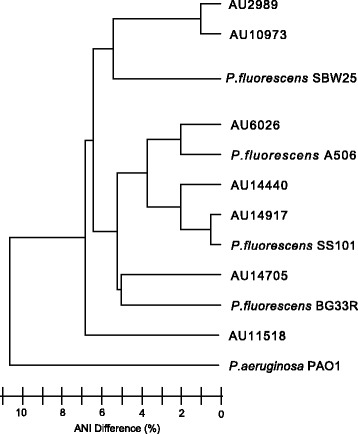
Phenogram based on average nucleotide identity (ANI) between subclade III strains. Phenogram created based on a similarity matrix of the average nucleotide identity (ANI) between subclade III strains. Clustering performed using the Unweighted Pair Group Method with Arithmetic mean (UPGMA) analysis

Additional phylogenetic analysis of the clinical isolates was performed using average nucleotide identity (ANI) and unweighted pair group method with arithmetic mean (UPGMA) clustering (Fig. 2 and Additional file 3: Table S3). ANI uses the shared nucleotide identity between bacteria genomes to infer taxonomic relationships, where 95 % is considered the cut-off for species delineation [14, 16]. The percent ANI shared between members of subclade III ranged from 86 to 98.9 % (Additional file 3: Table S3). The ANI between *P. aeruginosa* PAO1 (a *Pseudomonas sp.* not found in the *P. fluorescens* species complex*)* and every *P. fluorescens* subclade III strain was below 79 %. Both of these numbers corresponded to previously determined intra- and inter-species shared ANI [4]. In summary, both MLSA and ANI analysis clearly revealed that each of the clinical strains belong within subclade III.

### Secondary metabolites

Members of the *P. fluorescens* species-complex are well-documented to produce numerous secondary metabolites necessary for living on plants and in the soil and rhizosphere [3]. The genetic sequence of every gene known to be involved in the production of the secondary metabolites listed in Table 3 was used to screen the newly sequenced clinical *P. fluorescens* strains using the standalone BLASTn function provided by NCBI [17] (Additional file 4: Table S4). The panel of genes involved in the production of secondary metabolites found in the clinical strains agrees with what has been reported from previously sequenced *P. fluorescens* subclade III strains, in terms of presence, absence and variable presence [3]. None of the strains screened contained the genes necessary to make any of the seven *P. fluorescens*-associated antibiotics (Table 3). In contrast, every strain contained the full gene cluster to produce the siderophore pyoverdine [18, 19] (Table 3). In addition to the major siderophore pyoverdine, *P. fluorescens* strains produce multiple secondary siderophores. The genes necessary to produce the secondary siderophore, pseudomonine, were found in clinical strain AU14705 and environmental strains A506 and BG33R. Another secondary siderophore, hemophore, is involved in the chelation of heme from eukaryotic hosts [20]. The gene cluster required for the biosynthesis and efflux of this siderophore was found in clinical strains AU14705 and AU14917 and environmental strains SS101 and BG33R1. The environmental strains A506, SS101 and BG33R1 are all known to produce chitinase [3] (Table 3). Three clinical strains, AU6026, AU14705 and AU14917, also contained the gene cluster necessary to produce this enzyme. Every member of subclade III contained all four genes of the exoprotease AprA gene cluster. Our analysis revealed that clinically derived subclade III strains contain the secondary metabolite gene clusters known to be present in environmentally-derived subclade III strains.

**Table 3:** Secondary metabolite genes and gene clusters in subclade III strains

		Antibiotics							Siderophores					Exoenzymes			Plant-Bacterial Communication						
		DAPG	HCN	Phenazine	Pyrrolnitrin	Rhizoxins	Pyoluteorin	Mupirocin	Pyoverdine	Pseudomonine	Enantio-pyochelin	Achromobactin	Hemophore	Chitanse	AprA	Pectate Lyase	IAA Biosynthesis	IAA Catabolism	PAA catabolism	ACC deaminase	2,3-bd biosynthesis	Acetoin catabolism (LP)	Acetoin catabolism (DP)
Human-associated Isolates	AU2989								X						X								X
	AU6026								X					X	X								X
	AU10973								X						X								
	AU11518								X						X								X
	AU14705								X	X*			X	X	X								
	AU14917								X				X	X	X								
Environment-associated Isolates	SBW25 UM								X						X	X							
	*P.fluorescens* SBW25								X						X	X							
	*P.fluorescens* A506								X	X				X	X								X
	*P. fluorescens* SS101								X				X	X	X								X
	*P. synxantha* BG33R1								X	X*			X	X	X			X					

The presence or absence of a gene or gene cluster within each sequenced genome. The nucleotide sequence for the gene(s) involved in the biosynthesis of each secondary metabolite was used to query each genome with the BLASTn function provided by NCBI. The chosen query nucleotide sequence for each gene is detailed in Additional file 4: Table S4. A ‘Yes’ destination corresponds to a BLASTn hit with an e value <1×10^15^ and query and sequence identity >70%. The asterisk (*) indicates that one gene within the gene cluster fell below the cutoff

### Pan, accessory, core genomes

**Fig. 3:**
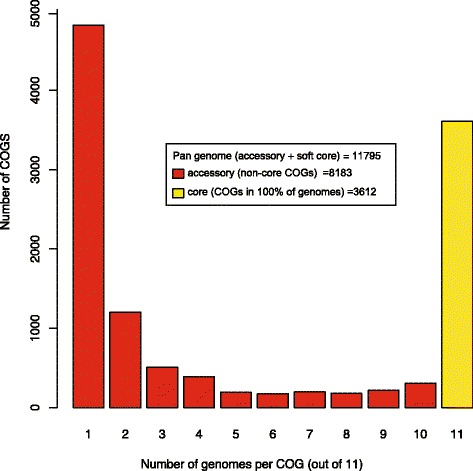
The pan, accessory and core genomes in subclade III strains. Each of the 11795 COGS was analyzed to determine how many genomes encoded that particular COG (1–11, x-axis). Then the number of COGS encoded by only one genome, two genomes, etc. was determined (y-axis). The pan genome contains the accessory and core. The average number of COGs per genome is 5592 (range: 5332–6123). The pan genome and its compartments was calculated using the COGtriganle clustering algorithm in GET_HOMOLOGUES [82, 83]

**Fig. 4:**
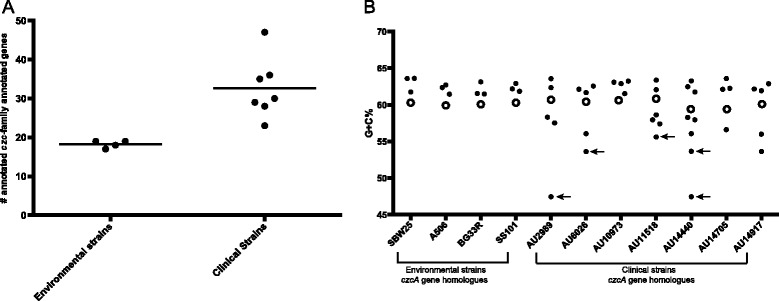
The *czc* gene cluster in environmental and clinical subclade III strains. A. The total number of genes annotated as belonging to the *czcA* gene cluster in environmental and clinical subclade III strains. B. The percent G + C content of the *czcA* gene homologues. Open circles correspond to the G + C content found across the entire genome. Open circles correspond to the G + C content found across the entire genome. Arrows indicate the *czcA* homologs enclosed in boxes in Fig. 5

To determine the pan, accessory, and core genomes of the subclade III *P. fluorescens* strains, we calculated the number of clusters of orthologous groups of proteins (COGS) shared among the strains. On average, the strains in our study contained 5592 COGs per genome, with a range of 5332–6123. The pan genome, which includes all COGs, shared and unshared, among all the genomes, consisted of 11,795 individual COGs (Fig. 3). The core genome, which refers to only those COGs that are shared between all genomes, contained 3612 COGs (Fig. 3). The accessory genome, which is all the COGs not part of the core, contained 8183 individual COGs. Comparison of the genomes of the clinical strains and the environmental isolates revealed that they share the same general assortment of COGs. Clinical strains were found to have additional copies of COGs involved in metal-resistance, in particular, those of the *czc*-gene family (Fig. 4a). Proteins produced through the *czc* gene cluster are important in regulating resistance to potentially toxic metals, such as zinc, copper, cobalt and cadmium, in gram-negative bacteria [21–25]. Though members of the *czc* gene family were also found in environmental strains, clinical strains had up to twice as many members of this gene family per genome (Fig. 4a). This suggested that either duplication or genetic transfer of the metal-resistance gene cluster occurred recently in the clinical strains. While the identity of the conserved protein families was the same across the subclade III genomes, COGs involved in metal resistance were found in significantly higher numbers in the clinical strains.

### Metal resistance genes

**Fig. 5:**
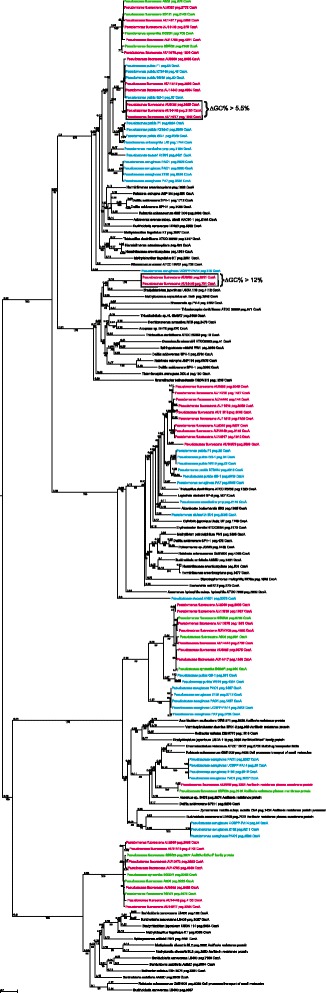
Neighborhood-joining phylogenetic tree based on the amino acid sequence of *czcA* homologues. *czcA* homologues from clinical strains are highlighted in red; homologs from environmental strains are highlighted in green; homologues from other *Pseudomonas spp*. are highlighted in blue. Amino acid sequences of the proteins expressed from *czcA* homologues discovered in RAST [78]. All other bacteria species are publically available on RAST. The amino acid sequences were aligned with MAFFT [80, 81]

Environmental strains contained on average 18 genes belonging to the *czc* gene cluster (range 17–19), while clinical strains contained on average 33 *czc*-related genes (range 23–47). The *czcCBA* gene cluster encodes for a resistance nodulation cell division (RND)-type efflux pump that actively shuttles the metal cations out of the bacterial cell [26, 27]. The expressed CzcA protein constitutes the inner part of the of the RND efflux pump, and is a chemiosmotic cation/proton antiporter driven by a H+ gradient [26, 27]. Focusing on just the gene that encodes for the CzcA protein, environmental strains contained three *czcA* homologues each, while clinical strains contained between three and eight *czcA* homologues (Fig. 4a). To determine whether the presence of additional copies of the *czcA* gene was due to recent gene acquisition, the GC content across each *czcA* homologue was compared to the GC content across the entire genome of each strain. In the environmental strains, the *czcA* homologues had had similar GC content as that found across the entire genome (Fig. 4b). In contrast, in the clinical strains, there were *czcA* homologues that had significantly lower GC content than that found across the entire genome. Three *czcA* homologues (from AU6026, AU11518 and AU14440) had more than a 5.5 % difference in GC content when compared to the entire genome, while two *czcA* homologues (from AU2989 and AU14440) had more than a 12 % difference in GC content (Figs. 4b and 5). This level of difference in GC content suggests that these *czcA* gene clusters could represent recent events of horizontal gene transfer.

To further investigate whether the clinical strains gained additional *czcA* homologues through horizontal gene transfer, we built a phylogenetic tree containing the predicted amino acid sequences of the expressed CzcA proteins from clinical and environmental subclade III strains, as well as the amino acid sequences of representative *Pseudomonas* and non-*Pseudomonas* strains (Fig. 5). Each clinical strain had three CzcA homologues that grouped with the three CzcA homologues in the environmental strains (Fig. 5). Additionally, the clinical strains contained a CzcA protein whose most similar neighbor was the CzcA protein from *P. putida*. The proteins expressed from *czcA* gene homologues with significantly lower GC content are labeled on the tree. The three CzcA proteins expressed from genes with a GC content difference of more than 5.5 % all grouped together in the tree, and are most closely related to the CzcA proteins from *P. putida* strains F1, KT2440 and GB-1, as well a CzcA protein from *P. fluorescens* A11518 and AU14440. The CzcA proteins expressed from *czcA* genes with more than 12 % difference in GC content did not branch off with any CzcA homologue from a *Pseudomonas* strain, but instead, branched with a protein from *Bradyrhizobium japonicum* USDA110 (Fig. 5), further suggesting that these gene copies were acquired through recent horizontal gene transfer. Clinical subclade III strains contain homologues of *czcA* that show evidence of recent horizontal transfer from non-*Pseudomonas* bacteria strains.

### GC islands

We performed total and segmented GC content scans across the genomes of the newly sequenced clinical subclade III strains to look for genomic regions of recent horizontal gene transfer. The range in total GC content of the newly sequenced clinical strains (59.5 to 60.8 %) was similar to the range seen in the previously sequenced environmental strains (59.6 to 60.5 %) (Table 2). However, the segmented GC scans revealed genomic regions where the GC content dropped drastically from the overall average (Additional file 5: Figure S1). We referred to the regions of lower GC content as Lower GC Islands (LGCI) and those with more than a 15 % difference from the overall genome GC average were selected for further investigation. We used the coordinates of the LGCIs provided by the GC-Profile tool to select the nucleotide sequence of the LGCI from each isolate’s genome. This nucleotide region was then used to screen the non-redundant nucleotide database on NCBI with the online megablast function (http://blast.ncbi.nlm.nih.gov/Blast.cgi). The results of the LGCI nucleotide screens are presented in Additional file 6: Table S5. The nucleotide screens revealed that some of the LGCIs are more genetically similar to *Pseudomonas* species outside of the *P. fluorescens* species-complex, suggesting regions of recent horizontal gene transfer into the clinical strains.

### The PfiT/I2 region

**Fig. 6:**
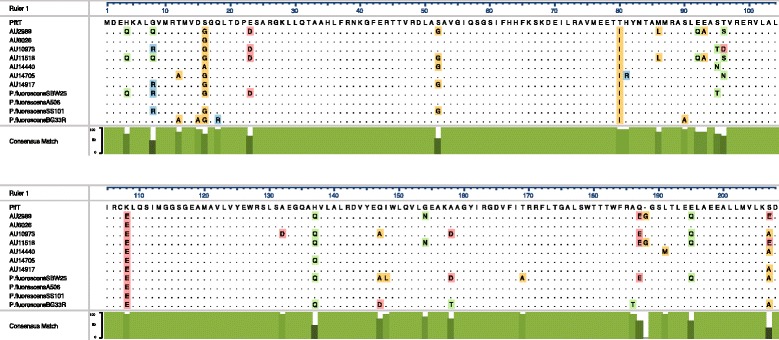
Alignment of the PfiT amino acid sequences from subclade III strains. Amino acid sequence of the PifT protein aligned using MUSCLE [87]. Published sequence of PfiT used as reference (labeled PfiT) [27]. Only amino acids differing from reference are shown and colored. Percent nucleotide consensus match visualized in green underneath alignment. Alignment visualized with DNAstar MegAligPro

Although PfiT/I2 has been identified as a *P. fluorescens-* specific immunostimulatory protein, no one has yet characterized the PfiT/I2 sequence in the genomes of fully sequenced *P. fluorescens* strains. The PfiT/I2 region of *P. fluorescens* encodes for an antigenic peptide (I2), which has superantigen properties and is highly associated with various enteric and autoimmune diseases [28]. To investigate differences at the amino acid level, we screened each subclade III strain using a local BLASTp search with the published PfiT amino acid sequence [29] and the top hit was selected from each genome to create a multiple protein sequence alignment (Fig. 6). The original published *P. fluorescens* PfiT protein sequence was used as a reference and the residues that differ from the reference were highlighted in the figure. Just 29 of the 208 amino acids (13.9 %) showed any variation between the strains. Seven of these amino acid changes were seen in just one of the subclade III strains. Most notably, two residues were altered in every strain: a threonine to an isoleucine at position 80 and a lysine to a glutamic acid at position 108. The PfiT protein was previously determined to belong to the TetR-family transcriptional regulators and none of the substitutions that occurred in the PfiT sequence from subclade III strains occurred in residues known to be important for DNA binding [28]. Thus, PfiT/I2 is highly conserved at the amino acid level in all the *P. fluorescens* strains analyzed in our study.

### Secretion systems

#### Type II secretion system

**Fig. 7:**
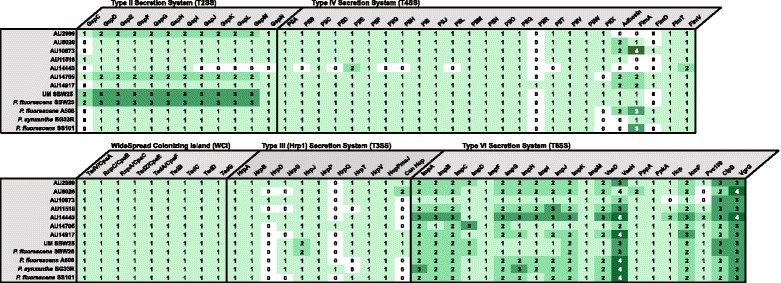
Genes that encode the Type-II, III, IV, VI and WCI secretion system in subclade III strains. Genes annotated using the RAST pipeline [77]. Full gene names listed in Additional file 7: Table S6

The Type II secretion system (T2SS), also known as the general secretion (gsp) pathway, is a highly conserved secretion system within Gram-negative bacteria and is prevalent among gamma-proteobacteria [30, 31] and the *Pseudomonas* genus [32]. The T2SS contains twelve core components: an outer membrane secretin (GspD); a cytoplasmic ATPase (GspE); an inner membrane protein (GspF); major (GspG) and minor (GspH, I, J, K) pseudopilins, proteins that assist with ATPase attachment to the inner membrane and form an inner membrane platform (GspL, M); a pre-pseudopilin peptidase/methyltransferase (GspO) and a protein possibly involved in substrate recognition or secretion interactions (GspC) [30, 33]. Gamma-proteobacteria can contain either one [30], or multiple [34–36], set(s) of T2SS genes (*gspCDEFGHIJKLMO*). Every subclade III strain but AU14440 contained between one and three T2SS gene clusters (Fig. 7 and Additional file 7: Table S6). These results agreed with what has been previously reported for T2SS gene clusters in subclade III strains [3]. Clinical strain AU14440 was missing five general secretion genes (*gspJKLMN*). These missing genes are necessary for the standard pilus structure of the T2SS [30, 33] and, therefore, AU14440 likely does not contain the standard type-II secretion system found in other *P. fluorescens* strains.

#### Type III secretion system

Type-III secretion systems (T3SS) are needle-like structures that bacteria use to deliver effector proteins directly into nearby host cells [37]. T3SSs fall into multiple families based on the genetic structure and the macromolecular structure of the needle complex. The Hrp1 T3SS [38] is the most common T3SS found in the *P. fluorescens* species-complex [3, 39–42]. The environmental subclade III strains SBW25, BG33R, A506, SS101, and the subclade II strains Q8r1-96 and Q2-87, all contained at least one copy of a Hrp1 family T3SS [3]. However, this system was not found in all *P. fluorescens* strains, as both Pf0-1 and Pf-5 did not have an Hrp1 T3SS gene cluster [3, 43]. A list of the genes annotated as belonging to the Hrp1 T3SS in the subclade III *P. fluorescens* strains is reported in Fig. 7**,** with the full annotated gene name shown in Additional file 7: Table S6. The genes *hrpD*, *hrpG*, *hrpQ* and *hrpT* all vary in whether or not they are included in the Hrp1 T3SS gene cluster. Environmental strain SBW25 has been reported to contain a functional T3SS [44], even though our analysis shows that it lacks *hrpD* and *hrpQ*, suggesting these two genes are not required for T3SS function. Whether or not HrpG and HrpT are required cannot be concluded until the functionality of a T3SS in additional subclade III strains is tested. Genes related to the Hrp1 T3SS were found in each of the subclade III strains analyzed. In summary, this is the first report demonstrating that the genomes of clinically-isolated strains of *P. fluorescens* encode for a T3SS.

#### Type-IV secretion system

In *P. aeruginosa*, the T4SS genes *pilABCDEFGHIJKLMNOPQRSTUVWXY1Y2Z* are required for twitching motility [45] and the subclade III strains analyzed here were missing between one and seven of the T4SS genes required for twitching motility in *P. aeruginosa* (Fig. 7 and Additional file 7: Table S6). The Type-IV secretion system (T4SS), also known as the Type-IV pili or fimbria, is a three-dimensional filament on the surface of bacteria that is important in adhesion, protease secretion and motility [46]. As was seen in the T2SS gene cluster (Fig. 7 and Additional file 7: Table S6), AU14440 was missing the largest number of T4SS-associated genes, lacking homologues to *pilBDGHLRT* and the genes that encode for the adhesins, FimA and FimD. Due to the large number of T4SS gene homologues missing in AU14440, it is likely that this isolate does not contain a functional T4SS. The number of gene homologues for the T4SS proteins adhesin, FimA and FimD, varied considerably between the subclade III strains. FimA, a type IV fimbria major subunit protein, shows the largest variation in gene homologues per genome, ranging from none (AU2989, AU11518 and AU14440) to four per genome (AU10973). Thus, every clinical subclade III strain contained a majority of the genes known to be required for twitching motility in *P. aeruginosa*, with the strain AU14440 missing the highest number of T4SS related genes.

We further analyzed the *fimA* genes from a sample group of subclade III strains (Additional file 8: Figure S2). We aligned the genetic sequences of *fimA* from A506, AU6026 and AU10973 using the MAFFT algorithm (Additional file 8: Figure S2). The alignment revealed that the genes annotated as *fimA* fall into two major groups: *fimA* ‘a’ group and *fimA* ‘b’ group. Subclade III strains A506 and AU10973 contained *fimA* homologues in both these two genetic groups. In addition, A506 and AU10973 both contained *fimA* homologues that fell within the same group but varied by single point mutations. This analysis revealed that *P. fluorescens* subclade III strains contained multiple homologues of the *fimA* gene and homologues can vary by point mutations along the sequence. As the *fimA* gene has only been annotated in a select number of *Pseudomonas spp.* (http://www.pseudomonas.com/) it is not possible to know if this level of redundancy is seen across the *P. fluorescens* species-complex or the rest of the *Pseudomonas* genus. Further analysis is required to understand the function of multiple *fimA* genes in the subclade III strains.

### Widespread Colonizing Island (WCI) / *tad* locus

The WCI has not previously been studied in *P. fluorescens.* Due to its importance in colonizing new environmental niches, the locus containing the *tad* genes is referred to as the ‘widespread colonization island’ (WCI) [47]. The *tad* locus is a newly described subtype of type II secretion system [48]. Proteins expressed by the *tad* locus are important for biofilm formation and colonization of hosts in multiple bacterial species. In *P. aeruginosa,* the genes within the WCI are regulated by the same quorum sensing circuit that regulates multiple virulence factors [49, 50]. All subclade III strains contained the ten WCI-associated genes: *cpaABCE* and *tadABCDG* (Fig. 7). Based on the presence and order of the genes in the *tad* locus, the *P. fluorescens* subclade III strains analyzed in this study can be predicted to encode a functional WCI.

### Type VI secretion system

Here we surveyed the Type- IV secretion system (T6SS) across the subclade III strains, and found that each strain carried between 1–3 T6SS gene clusters (Fig. 7). The T6SS is a macromolecular complex that either transports bacterial effectors directly into target cells or releases them into the extracellular medium [51]. Many of the T6SS genes found in the *P. fluorescens* strains are highly conserved across all bacterial strains that contain a T6SS [52] (Fig. 7 and Additional file 7: Table S6). Every *P. fluorescens* strain within this analysis contained at least one copy of *impABCDGHIJK*, *vasDH*, *ppA*, *ppK*A, *dotU*, *icmF*, *clpB*, *clpV1*, *vgrG1* and *vgrG2* genes. It has been reported that at least 11 structural proteins and two effector proteins (mainly Hcp and VgrG) are required to assemble a functional T6SS [52, 53]. Each subclade III strain also contained multiple copies of the effector proteins Hcp and VgrG. Multiple copies of effector proteins are thought to be tied to the multiple utilizations of the T6SS within a bacterial cell, allowing a bacterium to differently utilize the T6SS depending on environmental conditions [53]. The newly- sequenced human-associated subclade III strains contained multiple copies of the structural T6SS genes, and a large number of T6SS effectors, similar to what has been reported in other *P. fluorescens* strains.

The number of *vasH* homologues annotated in the subclade III strains ranged from two (AU10973 and AU14705) to five (AU14440). VasH is a sigma-54 dependent transcriptional regulator that has been shown to regulate transcription of the T6SS [54–56]. The sigma factor 54 is an alternate sigma factor that functions as a global regulator in multiple plant and animal associated bacteria [57, 58]. Sigma factor 54 interacts with RNA-polymerase and a sigma-54 dependent transcriptional regulator (such as VasH) to initiate transcription at sigma-54 dependent promoters [58, 59]. In order to investigate the differences between these *vasH* gene homologues, we did a multiple sequence alignment of the *vasH* genes from A506, AU6026, AU10973 and AU14440 and assembled a phylogenetic tree (Additional file 9: Figure S3). The separate *vasH* homologues fell into four separate groups based on nucleotide sequence similarity, labeled as groups ‘a’, ‘b’, ‘c’ and ‘d’. The *vasH* ‘a’ homologues showed the highest similarity to the *vasH* from *Vibrio cholera*. AU14440 also contained an additional copy of a *vasH* homologue that branches with the *Vibrio cholera vasH*. AU10973 also has two copies of a *vasH* homologue, one in branch ‘c’, and one in branch ‘d’. This analysis revealed that the different homologues of *vasH* fell within distinct evolutionary groups based on nucleotide similarity comparison.

## Conclusions

We present the first comparative genomic analysis of *Pseudomonas fluorescens* isolates from clinical samples. Previously, all sequenced *P. fluorescens* strains had been isolated from environmental samples, such as the soil, plant leaves and loam [1, 3]. Comparing the genome of seven strains of *P. fluorescens* isolated from the lungs of individuals with CF (Table 1) to four previously sequenced environmental strains within the same subclade (III), we found that all eleven strains were very similar in regard to global and individual genomic features (Tables 2 and 3; Fig. 6). However, the clinical strains analyzed in this study differed from the environmental strains in two important ways. Firstly, the strains isolated from clinical samples had an increased temperature growth range that allowed them to grow between 32 °C and 37 °C, while the representative environmental strain SBW25 could not grow above 28 °C. The finding that the clinical isolates studied in this paper can grow at 32 °C or higher agrees with other accounts of strains of *P. fluorescens* isolated from mammalian samples [5–8]. Changes in temperature often signal a change in environmental context, and bacteria have multiple mechanisms to sense these changes, including signaling through two-component sensor (TCS) systems [60]. It remains to be determined whether differences in a specific TCS may account for this difference.

The clinical strains also differed significantly from environmental strains in the number of protein-coding genes involved in the resistance nodulation cell division (RND)-type of efflux pump that regulates zinc, cadmium and cobalt ions in many gram-negative bacteria [24, 26, 61]. Efflux pumps control the concentration of metal ions that pass through a bacterial cell and there is strong evidence for a link between resistance to metal ions and antibiotics [61, 62]. Metals, such as zinc, are necessary for bacterial growth in trace amounts. However, when in abundance, metal cations become toxic to the bacterial cell. Zinc will bind to free thiol groups, which can disrupt protein function [63]. The RND-type efflux pump produced by the CzcCBA proteins is regulated by the CzcR-S two-component system [26]. In *P. aeruginosa*, treatment with zinc leads to the expression of the *czcRS* operon, which then leads to transcriptional activation of *czcCBA* [61]. There is a link between the metal resistance conferred through the *czcCBA* efflux pump and resistance to antibiotics, such as imipenem. The same two-component regulator that turns on transcription of *czcCBA* also negatively regulates the gene *oprD*. This gene encodes for the specific porin, OprD, which is the primary route by which carbapenems, such as impienem, enter into a bacterial cell [62]. In laboratory conditions, exposure to zinc can lead to spontaneous mutations in the *czcS* sensor gene in *P. aeruginosa,* making the bacteria resistant to imipenem exposure [61]. Increased resistance to antibiotics has been seen in multiple different metal-contaminated environments, such as freshwater streams [64], costal areas [65], and metal-contaminated ash settling basins [66]. GC content and phylogenetic analysis of the *czcA* gene involved in zinc, cadium and cobalt resistance suggested that clinical strains obtained additional copies through horizontal gene transfer from bacteria outside of the *Pseudomonas* genus.

Our findings that the clinical strains were highly similar to environmental strains within the same phylogenetic subclade is in itself significant because it highlights that many of the qualities that *P. fluorescens* bacteria utilize to succeed in the environment also benefit survival in a human host. Environmental and clinical strains of *P. aeruginosa* are often very similar genomically [67, 68], with a few notable differences that include increased temperature growth range [69] and the acquisition of additional antibiotic resistance traits [70–74]. The clinical strains analyzed in our current study all fell into subclade III of the *P. fluorescens* species complex; further studies will likely that reveal clinical strains of *P. fluorescens* in each of the phylogenetic divisions. The very definition of an “environmental”, “clinical” or “human” strain is in fact an artificial construct; bacteria will colonize and persist in any niche that provides the requirements necessary for growth. As more clinical strains of *P. fluorescens* are discovered, sequenced and analyzed, our understanding of what genetic attributes are important for the various lifestyles of *P. fluorescens* in its different niches is likely to expand and become more refined.

## Methods

### Strain isolation, storage and 16S typing

The seven *P. fluorescens* strains were isolated between March 2001 and January 2008, from five hospitals across the United States (Hartord, CT; Seattle, WA; Salt Lake City, UT; Little Rock, AR; Augusta, GA, Table 1)*.* The majority (six strains) were isolated from sputum; one strain was isolated from a throat swab. One strain was isolated from an infant with CF. *P. fluorescens* SBW25 was kindly provided by Dr. Stuart Levy (Tufts University). Isolates were banked and stored at -80C. The universal primer set 8 F and 1492R [75] was used to amplify a portion of the 16S rRNA gene through PCR, which was then Sanger-sequenced using an ABI 3730XL sequencer. Partial 16S sequences were screened through NCBI’s nonredundant nucleotide database and identified as *P. fluorescens.* Individual strains were either streaked out on LB agar for phenotypic assays or grown up aerobically overnight in Luria Broth at 34 °C for DNA isolation.

### Phenotypic assays

To determine the temperature range for growth, a single colony was selected from an agar plate and grown in Luria Broth aerobically for 18 h at room temperature (22 °C), 34 °C and 37 °C. The optical density at 600 nm was measured and compared to non-inoculated broth. Plates were incubated at 34 °C for twenty-four hours and colony forming were counted and used to calculate doubling time.

All phenotypic assays were performed from a single colony grown up from an −80 °C glycerol stock maintained in the lab. Fluorescence was assayed using an UV light. Standard Gram-stain was used to determine whether each strain was gram-negative or positive. Motility was assayed by looking for movement on a wet mount under 100x magnification.

### DNA isolation

Genomic DNA was extracted from 2 mL of overnight culture growth using the DNeasy Blood and Tissue Kit (Qiagen Cat# 69506) following manufacturer’s recommendations.

### Genome sequencing, assembly and annotation

Whole genome sequencing was performed at the University of Michigan Sequencing Core on the Illumina HiSeq 2000 platform with a 100-bp paired-end library. Illumina reads were assembled *de novo* with the DNAstar SeqMan NGen Version 12 software. The seven genomes were assembled into contigs and contigs were ordered with the Mauve aligner ‘reordering contigs’ function [76] using a previously sequenced *P. fluorescens* subclade III genome (A506) as a reference. Annotation was performed using the automated online software RAST [77, 78]. The presence or absence of secondary metabolite genes was assayed through the BLASTn function provided by NCBI [17] using a chosen query sequence listed in Additional file 4: Table S4 and a cutoff of evalue <1e15 and sequence identity >70 % [79].

### Nucleotide sequence accession numbers

The draft genomes have been deposited at DDBJ/EMBL/GenBank with accession numbers JRXT00000000, JRXU00000000, JRXV00000000, JRXW00000000, JRXX00000000, JRYA00000000, JRXY00000000, for isolates AU2989, AU6026, AU10973, AU11518, AU14440, AU14705, AU14917, respectively.

### MLSA and phylogenetic analysis

Multi-locus sequence analysis was performed using the concatenated sequences of the following housekeeping genes- *dnaE*, *ppsA*, *recA*, *rpoB*, *guaA*, *mutL*, *pyrC* and *acsA*- modified from the approach in Loper et al. [3]. The nucleotide sequences of the *czcA* homologues were discovered in RAST and used to infer a phylogenetic tree. The sequences used to construct the MLSA and *czcA* homologue phylogenetic trees were aligned using MAFFT [80, 81] and the trees were inferred using a neighborhood-joining method with 1000 bootstraps. Shared average nucleotide identity (ANI) was determined using the online software at http://enve-omics.ce.gatech.edu/ani [14]. A similarity matrix of the ANI between the subclade III strains was created and clustering performed using the Unweighted Pair Group Method with Arithmetic mean (UPGMA) algorithm.

### Pan, accessory and core genome and GC analysis

The pan, accessory and core genomes were calculated using the COGtriangle algorithm [82] in GET_HOMOLOGUES [83]. The COG algorithm detects candidate sets of orthologous proteins, with the given requirement that included proteins are also found in at least three evolutionarily distant species [84, 85]. Each protein represented by a COG is therefore thought to have evolved from a single ancestral gene [84]. GC island content and islands were calculated using the online GC profile tool at http://tubic.tju.edu.cn/GC-Profile/ [86]. The GC-Profile tool segments an inputted genome based on parameters provided by the user and provides the GC content across the genome segments, as well as the cumulative GC content of the entire genome.

### Analysis of individual genes

The amino acid sequence of PfiT from subclade III strains was aligned using MUSCLE [87]. Alignment of the f*imA* nucleotide acid homologues was performed using the MAFFT algorithm [80, 81]. The nucleotide sequences of *vasH* homologues were aligned in Mauve [88]. The PfiT, *fimA* and *vasH* alignments were visualized using DNAstar’s MegaAlignPro software.

### Availability of supporting data

The data sets supporting the results of this article are available in the NCBI whole genome repository at http://www.ncbi.nlm.nih.gov/genome/.

### Ethics statement

The bacterial samples in this study were exempt from ethics approval: they were provided by clinical microbiology labs, as de-identified cultures, with all patient identifiers removed.

## Additional files

Additional file 1: Table S1. Assembly statistics of the *P. fluorescens* strains in this study. The paired end reads from Illumina HiSeq were *de novo* assembled using the DNAstar SeqMan NGen. (PDF 387 kb) Additional file 2: Table S2. The nucleotide sequence of the 16S rRNA sequences was used to query the NCBI nucleotide collection (nr/nt). (PDF 170 kb) Additional file 3: Table S3. Average nucleotide identity (ANI). Shared average nucleotide identity between each strain in subclade III was determined using the online tool at http://enve-omics.ce.gatech.edu/ani [14]. Values presented in % shared ANI and ≥95 % shadowed in gray. (PDF 370 kb) Additional file 4: Table S4. Secondary metabolite genes and reference organism for BLAST. Each gene involved in the production of the secondary metabolites analyzed in Table 3 is listed in the left column. The source organism of each gene’s nucleotide sequence used for the blast search is in the middle column. The NCBI ID for each source organism is the right column. (PDF 54 kb) Additional file 5: Figure S1. GC content calculated using the online GC profile tool [86]. GC content displayed on y-axis and position in draft genome displayed on x-axis. Arrows indicate GC islands that have been further analyzed via NCBI BLASTn, results in Additional file 6: Table S5. (PDF 559 kb) Additional file 6: Table S5. BLAST results of selected GC Islands. The nucleotide sequences of selected GC Islands (indicated with arrows in Additional file 5: Figure S1) were used to query the NCBI nucleotide collection (nr/nt). The top six results are displayed. (PDF 48 kb) Additional file 7: Table S6. Full annotation of genes involved in secretion systems in subclade III strains. Abbreviations on left correspond to those found in Fig. 7 of paper. Fully annotated gene names are in the right column, as provided by the RAST annotation pipeline [78]. (PDF 47 kb) Additional file 8: Figure S2. Alignment of *fimA* homologues from select environmental and clinical subclade III strains. *fimA* homologues defined in RAST [78]. Nucleotide sequence of *fimA* homologues aligned with the MAFFT algorithm within DNAstar’s MegAlign Pro software. A506 is a representative environmental strain; AU6026 and AU10973 are representative clinical strains. (PDF 1267 kb) Additional file 9: Figure S3. Phylogenetic tree inferred from the nucleotide acid sequences of *vasH* homologues from representative environmental and clinical subclade III strains. Mauve algorithm used for alignment [88]. A506 is a representative clinical strain; AU6026, AU10973, AU14440 are representative clinical strains. (PDF 346 kb)

## Abbreviations

*P. fluorescens*: *Pseudomonas fluorescens*
CF: Cystic fibrosis
MLSA: Multi-locus sequence analysis
T3SS: Type III secretion system
UV: Ultra violet
ANI: Average nucleotide identity
UPGMA: Unweighted pair group method with arithmetic mean
COGS: Clusters of orthologoups groups
RND: Resistance nodulation cell division
GCI: GC Islands
T2SS: Type II secretion system
gsp: general secretion pathway
Hrp1: Hypersensitive response in plants
T4SS: Type IV secretion system
WCI: Widespread colonization island
T6SS: Type VI secretion system
