# Supplementary material for: Comparative genomics of Pseudomonas fluorescens subclade III strains from human lungs
Source: BMC Genomics. 2015 Dec 7;16:1032. doi: 10.1186/s12864-015-2261-2 (PMC4672498; doi:10.1186/s12864-015-2261-2)
Supplement: Additional file 1: Table S1. — Assembly statistics of the P. fluorescens strains in this study. The paired end reads from Illumina HiSeq were de novo assembled using the DNAstar SeqMan NGen. (PDF 387 kb) [file 12864_2015_2261_MOESM1_ESM.pdf]

Additional File 1. Assembly statistics of the *P. fluorescens* strains in this study.

		AU2989	AU6026	AU10973	AU11518	AU14440	AU14705	AU14917
**Assembly Totals**	# of Contigs	119	101	65	136	323	179	136
	Contigs >2K	64	70	42	66	238	42	82
	Assembled Sequences	3470296	2721507	3857173	3986627	3689916	4236424	4073286
	Unassembled Sequences	113880	64231	98101	177547	154426	309290	179064
	Sequences not assembled due to complete trimming	6672	5466	8507	7330	6450	9690	9423
	Sequences removed due to small contig size	44657	24398	28587	99733	74646	227149	83582
	All Sequences	3584176	2785738	3955274	416417	3844342	454714	4252350
	Contig N50	196 kbases	165 kbases	303 kbases	230 kbases	47 kbases	323 kbases	117 kbases
	Average Coverage	56	44	62	64	60	68	66
**Average Totals**	Sequences per Contig	29162	26945	59341	29313	11423	23667	29950
**Average Lengths**	Contigs	52308	60585	95565	46636	21338	34118	46707
	Assembled Sequences	97	97	97	97	97	97	97
	Unassembled Sequences	72	62	66	79	78	85	76
	All Sequences	96	96	96	96	97	96	96
**Average Quality**	Assembled Sequences	33	33	33	33	33	33	33
	Unassembled Sequences	27	27	26	29	29	30	29
	All Sequences	33	22	33	33	33	33	33
**Assembled Pair Statistics**	Read Pairs	1792088	1392869	1977637	2082087	1922171	2272857	2126175
	Assembled Pairs	1705204	1342761	1898009	1959906	1807056	2073494	2001733
	Pairs Consistent Within a Contig	1698401	1337220	1893522	1951647	1796379	2065932	1992757
	Pairs Inconsistent Within a Contig	247	256	60	163	56	87	100
