# Supplementary material for: Comparative genomics of Pseudomonas fluorescens subclade III strains from human lungs
Source: BMC Genomics. 2015 Dec 7;16:1032. doi: 10.1186/s12864-015-2261-2 (PMC4672498; doi:10.1186/s12864-015-2261-2)
Supplement: Additional file 2: Table S2. — The nucleotide sequence of the 16S rRNA sequences was used to query the NCBI nucleotide collection (nr/nt). (PDF 170 kb) [file 12864_2015_2261_MOESM2_ESM.pdf]

Additional File 2. The nucleotide sequence of the 16S rRNA sequences was used to query the NCBI nucleotide collection (nr/nt).

Megablast results based on A506 16S	Sequence Identity	Query Coverage	Gaps (%)
AU2989	98.56	90.04	0.29
	97.81	54.22	0.49
AU6026	99.61	67.61	0.00
	99.55	43.73	0.00
	99.67	39.97	0.00
	99.62	34.30	0.00
	100.00	11.74	0.00
AU10973	98.13	45.84	0.00
	97.69	37.14	1.07
AU11518	98.56	82.26	0.16
	97.98	49.08	0.27
	97.50	37.01	0.71
AU14440	99.90	66.62	0.00
	99.82	37.40	0.00
	99.82	36.28	0.00
	99.31	28.50	0.00
AU14705	98.73	99.01	0.00
	98.18	61.54	0.00
	98.09	51.78	0.00
	99.63	17.88	0.00
	93.90	14.05	0.94
AU14917	99.83	76.06	0.00
	99.91	69.53	0.00
	100.00	18.54	0.00
P. fluorescens SBW25	98.81	100.00	0.13
	98.81	100.00	0.13
	98.81	100.00	0.13
	98.81	100.00	0.13
