# Supplementary material for: Comparative genomics of Pseudomonas fluorescens subclade III strains from human lungs
Source: BMC Genomics. 2015 Dec 7;16:1032. doi: 10.1186/s12864-015-2261-2 (PMC4672498; doi:10.1186/s12864-015-2261-2)
Supplement: Additional file 3: Table S3. — Average nucleotide identity (ANI). Shared average nucleotide identity between each strain in subclade III was determined using the online tool at http://enve-omics.ce.gatech.edu/ani [14]. Values presented in % shared ANI and ≥95 % shadowed in gray. (PDF 370 kb) [file 12864_2015_2261_MOESM3_ESM.pdf]

**Additional File 3. Average nucleotide identity (ANI).**

| | AU2989 | AU6026 | AU10973 | AU11518 | AU14440 | AU14705 | AU14917 | SBW25 | SBW25_UM | A506 | SS101 | BG33R | PAO1 |
|---|---|---|---|---|---|---|---|---|---|---|---|---|---|
| AU2989 | 100 | | | | | | | | | | | | |
| AU6026 | 86.04 | 100 | | | | | | | | | | | |
| AU10973 | 97.75 | 86.75 | 100 | | | | | | | | | | |
| AU11518 | 86.45 | 86.22 | 86.46 | 100 | | | | | | | | | |
| AU14440 | 86.36 | 84.67 | 86.89 | 86.34 | 100 | | | | | | | | |
| AU14705 | 86.10 | 89.31 | 87.08 | 86.04 | 89.49 | 100 | | | | | | | |
| AU14917 | 86.12 | 94.82 | 86.81 | 86.08 | 96.15 | 89.42 | 100 | | | | | | |
| SBW25 | 88.42 | 88.66 | 91.77 | 88.39 | 88.84 | 89.14 | 88.86 | 100 | | | | | |
| SBW25 UM | 86.57 | 86.93 | 90.45 | 86.58 | 87.05 | 87.41 | 87.05 | 99.99 | 100 | | | | |
| A506 | 87.62 | 96.16 | 88.49 | 87.86 | 95.37 | 90.85 | 95.80 | 88.80 | 88.73 | 100 | | | |
| SS101 | 86.00 | 94.67 | 85.99 | 86.84 | 96.09 | 89.51 | 98.89 | 88.92 | 87.14 | 95.78 | 100 | | |
| BG33R1 | 85.82 | 89.51 | 86.72 | 85.88 | 89.45 | 89.75 | 89.61 | 88.70 | 86.93 | 91.10 | 89.69 | 100 | |
| PAO1 | 78.55 | 78.45 | 78.62 | 78.56 | 78.70 | 78.53 | 78.54 | 78.70 | 78.64 | 78.81 | 78.74 | 78.61 | 100 |
