# Supplementary material for: Comparative genomics of Pseudomonas fluorescens subclade III strains from human lungs
Source: BMC Genomics. 2015 Dec 7;16:1032. doi: 10.1186/s12864-015-2261-2 (PMC4672498; doi:10.1186/s12864-015-2261-2)
Supplement: Additional file 4: Table S4. — Secondary metabolite genes and reference organism for BLAST. Each gene involved in the production of the secondary metabolites analyzed in Table 3 is listed in the left column. The source organism of each gene’s nucleotide sequence used for the blast search is in the middle column. The NCBI ID for each source organism is the right column. (PDF 54 kb) [file 12864_2015_2261_MOESM4_ESM.pdf]

Additional File 4. Secondary metabolite genes and reference organism for BLAST.

Gene	Source Organism	NCBI ID
**DAPG**		
phlD	Pseudomonas fluorescens F113	NC_016830.1
**HCN**		
hcnA	Pseudomonas fluorescens Pf01	NC_007492.2
hcnB	Pseudomonas fluorescens Pf01	NC_007492.2
hcnC	Pseudomonas fluorescens Pf01	NC_007492.2
**Phenazine**		
phzA	Pseudomonas fluorescens S2P5	AY960782.1
phzB	Pseudomonas fluorescens S2P5	AY960782.1
phzC	Pseudomonas fluorescens S2P5	AY960782.1
phzD	Pseudomonas fluorescens S2P5	AY960782.1
phzE	Pseudomonas fluorescens S2P5	AY960782.1
phzF	Pseudomonas fluorescens S2P5	AY960782.1
phzG	Pseudomonas fluorescens S2P5	AY960782.1
**Pyrrolnitrin**		
prnA	Pseudomonas fluorescens Pf5	NC_004129.6
prnB	Pseudomonas fluorescens Pf5	NC_004129.6
prnC	Pseudomonas fluorescens Pf5	NC_004129.6
prnD	Pseudomonas fluorescens Pf5	NC_004129.6
**Rhizoxins**		
rhiA	Ralstonia solanacearum CFBP2957	NC_014307.1
rhiB	Ralstonia solanacearum CFBP2957	NC_014307.1
rhiC	Ralstonia solanacearum CFBP2957	NC_014307.1
rhiD	Ralstonia solanacearum CFBP2957	NC_014307.1
rhiE	Ralstonia solanacearum CFBP2957	NC_014307.1
rhiF	Ralstonia solanacearum CFBP2957	NC_014307.1
rhiG	Ralstonia solanacearum CFBP2957	NC_014307.1
rhiH	Ralstonia solanacearum CFBP2957	NC_014307.1
rhiI	Ralstonia solanacearum CFBP2957	NC_014307.1
**Pyoluteorin**		
pltM	Pseudomonas fluorescens Pf5	NC_004129.6
pltR	Pseudomonas fluorescens Pf5	NC_004129.6
pltL	Pseudomonas fluorescens Pf5	NC_004129.6
pltA	Pseudomonas fluorescens Pf5	NC_004129.6
pltB	Pseudomonas fluorescens Pf5	NC_004129.6
pltC	Pseudomonas fluorescens Pf5	NC_004129.6
pltD	Pseudomonas fluorescens Pf5	NC_004129.6
pltE	Pseudomonas fluorescens Pf5	NC_004129.6
pltF	Pseudomonas fluorescens Pf5	NC_004129.6

pltG	Pseudomonas fluorescens Pf5	NC_004129.6
pltZ	Pseudomonas fluorescens Pf5	NC_004129.6
pltI	Pseudomonas fluorescens Pf5	NC_004129.6
pltJ	Pseudomonas fluorescens Pf5	NC_004129.6
pltK	Pseudomonas fluorescens Pf5	NC_004129.6
pltN	Pseudomonas fluorescens Pf5	NC_004129.6
pltO	Pseudomonas fluorescens Pf5	NC_004129.6
pltP	Pseudomonas fluorescens Pf5	NC_004129.6
**Mupirocin**		
mupZ	Pseudomoans fluorescens NCIMB 10586	AF318063.3
mupA	Pseudomoans fluorescens NCIMB 10586	AF318063.3
mmpA	Pseudomoans fluorescens NCIMB 10586	AF318063.3
mupB	Pseudomoans fluorescens NCIMB 10586	AF318063.3
mmpB	Pseudomoans fluorescens NCIMB 10586	AF318063.3
mmpC	Pseudomoans fluorescens NCIMB 10586	AF318063.3
mmpD	Pseudomoans fluorescens NCIMB 10586	AF318063.3
mupC	Pseudomoans fluorescens NCIMB 10586	AF318063.3
macpA	Pseudomoans fluorescens NCIMB 10586	AF318063.3
mupD	Pseudomoans fluorescens NCIMB 10586	AF318063.3
mupE	Pseudomoans fluorescens NCIMB 10586	AF318063.3
macpB	Pseudomoans fluorescens NCIMB 10586	AF318063.3
mupF	Pseudomoans fluorescens NCIMB 10586	AF318063.3
macpC	Pseudomoans fluorescens NCIMB 10586	AF318063.3
mupC	Pseudomoans fluorescens NCIMB 10586	AF318063.3
mupG	Pseudomoans fluorescens NCIMB 10586	AF318063.3
mupH	Pseudomoans fluorescens NCIMB 10586	AF318063.3
mupJ	Pseudomoans fluorescens NCIMB 10586	AF318063.3
mupK	Pseudomoans fluorescens NCIMB 10586	AF318063.3
mmpE	Pseudomoans fluorescens NCIMB 10586	AF318063.3
mupL	Pseudomoans fluorescens NCIMB 10586	AF318063.3
mupM	Pseudomoans fluorescens NCIMB 10586	AF318063.3
mupN	Pseudomoans fluorescens NCIMB 10586	AF318063.3
mupO	Pseudomoans fluorescens NCIMB 10586	AF318063.3
mupP	Pseudomoans fluorescens NCIMB 10586	AF318063.3
mupQ	Pseudomoans fluorescens NCIMB 10586	AF318063.3
mupS	Pseudomoans fluorescens NCIMB 10586	AF318063.3
macpD	Pseudomoans fluorescens NCIMB 10586	AF318063.3
mmpF	Pseudomoans fluorescens NCIMB 10586	AF318063.3
macpE	Pseudomoans fluorescens NCIMB 10586	AF318063.3
mupT	Pseudomoans fluorescens NCIMB 10586	AF318063.3
mupU	Pseudomoans fluorescens NCIMB 10586	AF318063.3
mupV	Pseudomoans fluorescens NCIMB 10586	AF318063.3

mupW	Pseudomoans fluorescens NCIMB 10586	AF318063.3
mupR	Pseudomoans fluorescens NCIMB 10586	AF318063.3
mupX	Pseudomoans fluorescens NCIMB 10586	AF318063.3
mupI	Pseudomoans fluorescens NCIMB 10586	AF318063.3
**Pyoverdine**		
ppyR	Pseudomonas aeruginosa PAO1	NC_002516.2
pvdN	Pseudomonas aeruginosa PAO1	NC_002516.2
pvdE	Pseudomonas aeruginosa PAO1	NC_002516.2
pvdR	Pseudomonas aeruginosa PAO1	NC_002516.2
pvdT	Pseudomonas aeruginosa PAO1	NC_002516.2
pvdF	Pseudomonas aeruginosa PAO1	NC_002516.2
pvdG	Pseudomonas aeruginosa PAO1	NC_002516.2
pvdP	Pseudomonas aeruginosa PAO1	NC_002516.2
pvdO	Pseudomonas aeruginosa PAO1	NC_002516.2
pvdD	Pseudomonas aeruginosa PAO1	NC_002516.2
pvdJ	Pseudomonas aeruginosa PAO1	NC_002516.2
pvdS	Pseudomonas aeruginosa PAO1	NC_002516.2
**Pseudomonine**		
pmsC	Pseudomonas fluorescens	NC_017911.1
pmsE	Pseudomonas fluorescens	NC_017911.1
pmsA	Pseudomonas fluorescens	NC_017911.1
pmsB	Pseudomonas fluorescens	NC_017911.1
**Enantio-pyochelin**		
pchC	Pseudomonas fluorescens Pf-5	NC_004129.6
pchF	Pseudomonas fluorescens Pf-5	NC_004129.6
pchE	Pseudomonas fluorescens Pf-5	NC_004129.6
fetA	Pseudomonas fluorescens Pf-5	NC_004129.6
**Achromobactin**		
acsD	Pseudomonas syringae pv. phaseolicola 1448A	NC_005773.3
acsC	Pseudomonas syringae pv. phaseolicola 1448A	NC_005773.3
acsB	Pseudomonas syringae pv. phaseolicola 1448A	NC_005773.3
acsA	Pseudomonas syringae pv. phaseolicola 1448A	NC_005773.3
cbrA	Pseudomonas syringae pv. phaseolicola 1448A	NC_005773.3
crbB	Pseudomonas syringae pv. phaseolicola 1448A	NC_005773.3
crbC	Pseudomonas syringae pv. phaseolicola 1448A	NC_005773.3
crbD	Pseudomonas syringae pv. phaseolicola 1448A	NC_005773.3
**Hemophore**		
hasA	Pseudomonas protegens CHA0	NC_021237.1
**Chitanse**		
chiC	Pseudomonas fluorescens A506	NC_017911.1
**AprA**		
aprA	Pseudomonas fluorescens A506	NC_017911.1

**Pectate Lyase**		
pectate lyase	Pseudomonas fluorescens A506	NC_017911.1
**IAA Biosynthesis and Catabolism**		
iaaM	Pseudomonas protegens CHA0	NC_021237.1
iaaH	Agrobacterium tumefaciens	NC_002377.1
iacA	Pseudomonas putida 1290	NC_002947.3
iacC	Pseudomonas putida 1290	NC_002947.3
iacD	Pseudomonas putida 1290	NC_002947.3
iacE	Pseudomonas putida 1290	NC_002947.3
iacF	Pseudomonas putida 1290	NC_002947.3
iacG	Pseudomonas putida 1290	NC_002947.3
iacR	Pseudomonas putida 1290	NC_002947.3
iacH	Pseudomonas putida 1290	NC_002947.3
iacI	Pseudomonas putida 1290	NC_002947.3
**PAA catabolism**		
ppaX	Pseudomonas protegens Pf-5	NC_004129.6
paaY	Pseudomonas protegens Pf-5	NC_004129.6
paaB	Pseudomonas protegens Pf-5	NC_004129.6
paaD	Pseudomonas protegens Pf-5	NC_004129.6
paaF	Pseudomonas protegens Pf-5	NC_004129.6
paaG	Pseudomonas protegens Pf-5	NC_004129.6
paaH	Pseudomonas protegens Pf-5	NC_004129.6
paaI	Pseudomonas protegens Pf-5	NC_004129.6
paaJ	Pseudomonas protegens Pf-5	NC_004129.6
paaK	Pseudomonas protegens Pf-5	NC_004129.6
paaW	Pseudomonas protegens Pf-5	NC_004129.6
paaL	Pseudomonas protegens Pf-5	NC_004129.6
paaN	Pseudomonas protegens Pf-5	NC_004129.6
**ACC deaminase**		
acdS	Pseudomonas fluorescens F113	NC_016830.1
budC	Pseudomonas fluorescens A506	NC_017911.1
ydjL	Pseudomonas aeruginosa NCGM2.S1	NC_017549.1
**2,3-bd biosynthesis**		
ilvB	Pseudomonas fluroescens A506	NC_017911.1
ilvN	Pseudomonas fluroescens A506	NC_017911.1
**Acetoin catabolism (LP) and (DP)**		
acoR	Pseudomonas fluroescens A506	NC_017911.1
acoA	Pseudomonas fluroescens A506	NC_017911.1
acoB	Pseudomonas fluroescens A506	NC_017911.1
acoC	Pseudomonas fluroescens A506	NC_017911.1
acoX	Pseudomonas protegens Pf-5	NC_004129.6
bdhA	Pseudomonas protegens Pf-5	NC_004129.6
