# Supplementary figures and images for: Comparative genomics of Pseudomonas fluorescens subclade III strains from human lungs
Source: BMC Genomics. 2015 Dec 7;16:1032. doi: 10.1186/s12864-015-2261-2 (PMC4672498; doi:10.1186/s12864-015-2261-2)

## Additional File 5. GC content.

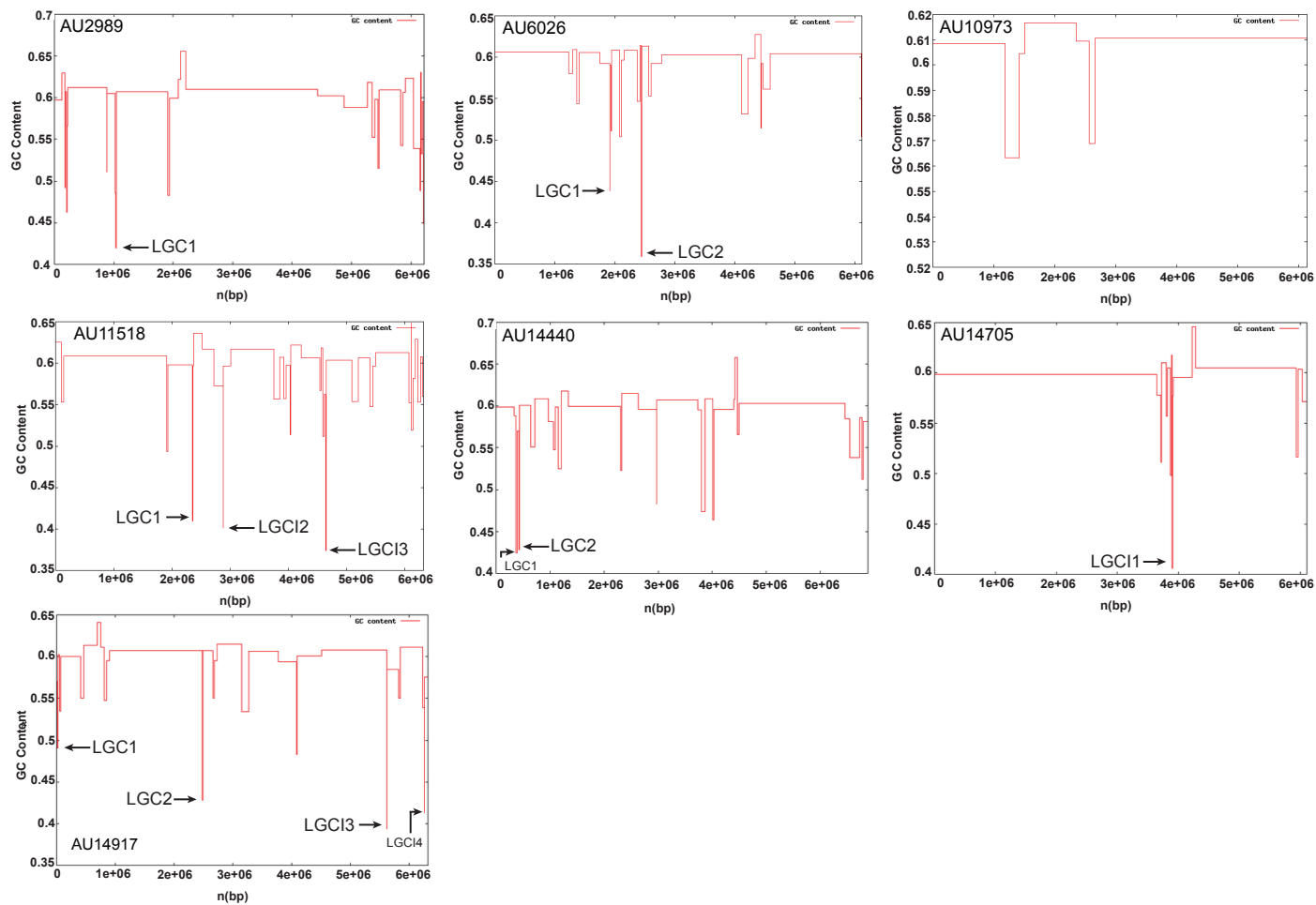

Supplement: Additional file 5: Figure S1. — GC content calculated using the online GC profile tool [86]. GC content displayed on y-axis and position in draft genome displayed on x-axis. Arrows indicate GC islands that have been further analyzed via NCBI BLASTn, results in Additional file 6: Table S5. (PDF 559 kb) [file 12864_2015_2261_MOESM5_ESM.pdf]
