# Supplementary material for: Comparative genomics of Pseudomonas fluorescens subclade III strains from human lungs
Source: BMC Genomics. 2015 Dec 7;16:1032. doi: 10.1186/s12864-015-2261-2 (PMC4672498; doi:10.1186/s12864-015-2261-2)
Supplement: Additional file 6: Table S5. — BLAST results of selected GC Islands. The nucleotide sequences of selected GC Islands (indicated with arrows in Additional file 5: Figure S1) were used to query the NCBI nucleotide collection (nr/nt). The top six results are displayed. (PDF 48 kb) [file 12864_2015_2261_MOESM6_ESM.pdf]

Additional File 6. Blast results of selected GC Islands.

Isolate	GC Island	GC(%)	Start	End	Size (nt)	Top Hits	E value	NCBI ID
AU2989	1	46.61	102157	127677	25520	Pseudomonas sp. TKP	0.00E+00	CP006852.1
						Pseudomonas sp. WCS374	5.00E-173	CP007638.1
						Pseudomonas fluorescens A506	5.00E-173	CP003041.1
						Pseudomonas fluorescens SBW25	1.00E-168	AM181176.4
						Pseudomonas simiae strain WCS417	3.00E-155	CP007637.1
						Pseudomonas fluorescens strain UK4	1.00E-138	3.00E-105
Isolate	GC Island	GC(%)	Start	End	Size (nt)	Top Hits	E value	NCBI ID
AU6026	1	44.58	1911471	1920380	8911	Pseudomonas sp. VLB120	0.00E+00	CP003961.1
						Pseudomonas alkylphenolia strain KL28	0.00E+00	CP009048.1
						Pseudmonas simiae strain WCS417	0.00E+00	CP007637.1
						Pseudomonas sp. WCS374	0.00E+00	CP007638.1
						Pseudomonas fluorescens A506	0.00E+00	CP003041.1
						Pseudomonas fluorescens SBW25	0.00E+00	AM181176.4
AU6026	2	37.5	2438921	2445960	7040	Pseudomonas fluorescesn A506	0.00E+00	CP003041.1
						Pseudomonas sp. WCS374	0.00E+00	CP007638.1
						Pseudomonas sp. TKP	2.00E-166	CP006852.1
						Pseudomonas simiae strain WCS417	2.00E-151	CP007637.1
						Pseudomonas fluorescens SBW25	5.00E-148	AM181176.4
						Pseudomonas poae RE*1-1-14	2.00E-136	CP004045.1
Isolate	GC Island	GC (%)	Start	End	Size (nt)	Top Hits	E value	NCBI ID
AU11518	1	41.9	2352011	2357520	4511	Pseudomonas fluorescens SBW25	6.00E-51	AM181176.4
						Pseudomonas sp. TKP	2.00E-45	CP006852.1
						Pseudomonas poae RE*1-1-14	1.00E-43	CP004045.1
						Pseudomonas fluorescens A506	5.00E-42	CP003041.1
						Pseudomonas sp. WCS374	1.00E-32	CP007638.1
						Pandoraea apista strain TF81F4	1.00E-27	CP010518.1
AU11518	2	45.56	2876941	287690	2751	Pseudomonas sp. VLB120	0.00E+00	CP003961.1
						Pseudomonas stutzeri DSM 4166	0.00E+00	CP002622.1
						Pseudomonas aeruginosa PA38182	0.00E+00	HG530068.1
						Klebesiella pneumoniae plasmid pKPN_CZ	0.00E+00	JX424424.1
						Enterobacter cloacae strain ECIH5 plasmid pENT-22e	0.00E+00	CP009855.1
						Enterobacter cloacae ECR091 plasmid pENT-4bd	0.00E+00	CP008907.1
AU11518	3	38.07	4641231	4643981	2750	Pseudomonas sp. TKP	1.00E-86	CP006852.1
						Pseudomonas sp. WCS374	8.00E-26	CP007638.1
						Pseudomonas fluorescens A506	8.00E-26	CP003041.1
						Pseudomonas fluorescens SBW25	2.00E-08	AM181176.4
						Pseudomonas simiae strain WCS417	1.00E-04	CP007637.1
Isolate	GC Island	GC (%)	Start	End	Size (nt)	Top Hits	E value	NCBI ID
AU14440	1	42.59	373671	390170	16501	Pseudomonas fluorescens A506	0.00E+00	CP003041.1

						Burkholderia pseudomallei TSV 48 chromosome 2	0.00E+00	CP009160.1
						Burkholderia pseudomallei NAU35A-3 chromosome 2	0.00E+00	CP004378.1
						Pseudomonas aeruginosa YL84	0.00E+00	CP007147.1
						Pseudomonas putida S12	0.00E+00	CP009974.1
						Pseudomonas sp. WCS374	0.00E+00	CP007638.1
AU14440	2	43.07	419101	430541	11440	Pseudomonas aeruginosa plasmid pUM505	0.00E+00	HM56097.1
						Pseudomonas fluorescens A506	0.00E+00	CP003041.1
						Pseudomonas aeruginosa PA38182	0.00E+00	HG530068.1
						Pseudomonas fluorescens SBW25	0.00E+00	AM181176.4
						Pseudomonas plecoglossicida strain NyZ12	0.00E+00	CP010359.1
						Pseudomonas aeruginosa PA96	0.00E+00	CP007224.1
Isolate	GC Island	GC (%)	Start	End	Size (nt)	Top Hits	E value	NCBI ID
AU14705	1	41.91	3897300	3900490	3190	Pseudomonas poae RE*1-1-14	9.00E-134	CP004045.1
						Pseudomonas fluorescens SBW25	3.00E-89	AM181176.4
						Pseudomonas sp. TKP	6.00E-79	CP006852.1
						Pseudomonas fluorescens A506	3.00E-76	CP003041.1
						Pseudomonas sp. WCS374	4.00E-75	CP007638.1
						Pseudomonas simiae strain WCS417	8.00E-52	CP007637.1
Isolate	GC Island	GC (%)	Start	End	Size (nt)	Top Hits	E value	NCBI ID
AU14917	1	39.52	1	1870	1871	Burkholderia ambifaria MC40-6 chromosome 2	2.00E-07	CP001026.1
AU14917	2	43.06	2480830	2489410	8691	Pseudomonas sp. WCS374	0.00E+00	CP007638.1
						Pseudomonas fluorescens A506	0.00E+00	CP003041.1
						Pseudomonas poae RE*1-1-14	5.00E-180	CP004045.1
						Pseudomonas simiae strain WCS417	3.00E-176	CP007637.1
						Pseudomonas fluorescens SBW25	7.00E-172	AM181176.1
AU14917	3	40.81	5618140	5620230	2200	Pseudomonas sp. TKP	1.00E-73	CP006852.1
						Pseudomonas resinovorans NBRC 106553	1.00E-73	AP013068.1
						Pseudomonas stutzeri CCUG 29243	1.00E-73	CP003677.1
						Pseudomonas stutzeri strain 36N1, ISPst11 TnpA (tnpA5) gene	1.00E-72	GQ221266.1
						Pseudomonas stutzeri strain	1.00E-72	EF648211.1
						Pseudomonas aeruginosa strain SG17M	1.00E-72	AF440524.1
AU14917	4	41.6	6256031	6262850	6820	Pseudomonas fluorescens A506	3.00E-143	CP003041.1
						Pseudomonas sp. WCS374	5.00E-134	CP007638.1
						Pseudomonas fluorescens strain UK4	4.00E-97	CP008896.1
						Pseudomonas putida 0CT plasmid alk genes cluster	7.00E-12	NG_035191.1
						Pseudomonas mandelii JR-1	2.00E-06	CP005960.1
						Pseudomonas brassicacearum subsp. brassicacearum NFM421	7.00E-06	CP002585.1
