# Supplementary material for: Comparative genomics of Pseudomonas fluorescens subclade III strains from human lungs
Source: BMC Genomics. 2015 Dec 7;16:1032. doi: 10.1186/s12864-015-2261-2 (PMC4672498; doi:10.1186/s12864-015-2261-2)
Supplement: Additional file 7: Table S6. — Full annotation of genes involved in secretion systems in subclade III strains. Abbreviations on left correspond to those found in Fig. 7 of paper. Fully annotated gene names are in the right column, as provided by the RAST annotation pipeline [78]. (PDF 47 kb) [file 12864_2015_2261_MOESM7_ESM.pdf]

Additional File 7. Full annotation of genes involved in secretion systems in subclade III strains.

	RAST Annotation
Type II Secretion	Type II Secretion
GspC	General Secretion Pathway protein C, GspC
GspD	General Secretion Pathway protein D, GspD
GspE	General Secretion Pathway protein E, GspE
GspF	General Secretion Pathway protein F, GspF
GspG	General Secretion Pathway protein G, GspG
GspH	General Secretion Pathway protein H, GspH
GspI	General Secretion Pathway protein I, GspI
GspJ	General Secretion Pathway protein J, GspJ
GspK	General Secretion Pathway protein K, GspK
GspL	General Secretion Pathway protein L, GspL
GspM	General Secretion Pathway protein M, GspM
GspN	General Secretion Pathway protein N, GspN
Type III Secretion (Hrp1)	Type III Secretion (Hrp1)
HrpA	Type III secretion pilin HrpA
HrpB	Type III secretion protein (Pto) HrpB
HrpD	Type III secretion protein HrpD
HrpG	Type II secretion protein HrpG
HrpJ	Type III secretion protein HrpJ
HrpP	Type III secretion protein HrpP
HrpQ	Type III secreiton component protein HrpQ
HrpT	Type III secretion protein HrpT
HrpV	Negative regulator of hrp expression HrpV
HopPmaJ	Type III effector HopPmaJ
Can Hop	Candidate Type III Hop effector
Type IV Secretion	Type IV Secretion
PIlA	Type IV pilin, PilA
PilB	Type II secretory pathway, ATPase PulE/Tfp pilus assembly pathway, ATPase PilB
PilC	Type IV fimbrial assembly protein, PilC
PilD	Type IV pilus biogenesis protein, PilD
PilE	Type IV pilus biogenesis protein, PilE
PilF	Type IV pilus biogenesis protein, PilF
PilG	Twitching motility protein, PilG
PilH	Twitching motility protein, PilH
PIlI	Type IV pilus biogenesis protein, PilI
PilJ	Type IV pilus biogenesis protein, PilJ
PIlL	Type IV pilus biogenesis protein, PilL

PilM	Type IV pilus biogenesis protein, PIlM
PIlN	Type IV pilus biogenesis protein, PIlN
PilO	Type IV pilus biogenesis protein, PilO
PIlQ	Type IV pilus biogenesis, PilQ
PilR	Type IV pilus biogenesis, PilR
PilT	Twitching motility protein PilT
PilV	Type IV fimbrial biogenesis protein, PilV
PIlW	Type IV fimbrial biogenesis protein, PIlW
PilX	Type IV fimbrial biogenesis protein, PilX
Adhesin	Adhesin major subunit pilin, adhesion
FimA	Type IV fimbria major subunit, FimA
FimD	Outer membrane usher protein, FimD
FimT	Type IV fimbrial biogenesis protein, FimT
FimV	Probably type IV pilus assembly FimV-related transmembrane protein
Widspread Colonizing Island (WCI)	Widspread Colonizing Island (WCI)
TadV/CpaA	Type IV prepillin peptidase,TadV/CpaA
RcpC/CpaB	Flp pilus assembly protein RcpC/CpaB
RcpA/CpaC	Type II/IV secretion system secretin RcpA/CpaC, assocaited with Flp pilus assembly
TadZ/CpaE	Type II/IV secretion system ATPase TadZ/CpaE, associated with Flp pilus assembly
TadA/CpaF	Type II/IV secretion system ATP hydrolase TadA/VirB11/CpaF, TadA subfamily
TadB	Flp pilus assembly protein TadB
TadC	Type II/IV secretion system protein TadC, associated Flp pilus assembly
TadD	Flp pilus assembly protein TadD, contains TPR repeats
TadG	Flp pilus assembly protein TadG
Type VI Secretion System	Type VI Secretion System
ImpA	Uncharacterized protein ImpA
ImpB	Uncharacterized protein ImpB
ImpC	Uncharacterized protein ImpC
ImpD	Uncharacterized protein ImpD
ImpF	Uncharacterized protein ImpF
ImpG	Protein ImpG/VasA
ImpH	Uncharacterized protein ImpH/VasB
ImpI	Uncharacterized protein ImpI/VasC
ImpJ	Uncharacterized protein ImpJ/VasE
ImpK	Uncharacterized protein ImpK/VasF/OmpA/MotB domain
ImpM	Protein phosphatase ImpM
VasD	Type IV secretion lipoprotein/VasD
VasH	Sigma-54 dependent transcriptional regulator
PppA	Phosphoprotein phosphatase PppA

PpkA	Serine/threonine protein kinase (EC 2.7.11.1) PpkA
Hcp	Secreted protein Hcp
IcmF	IcmF-related protein
Pvc109	Uncharacterized protein similar to VCA0109
ClpB	ClpB protein
VgrG	VgrG protien
