# Supplementary material for: Comparative genomics of Pseudomonas fluorescens subclade III strains from human lungs
Source: BMC Genomics. 2015 Dec 7;16:1032. doi: 10.1186/s12864-015-2261-2 (PMC4672498; doi:10.1186/s12864-015-2261-2)

Additional File 8. Alignment of *fimA* homologues from select environmental and clinical subclade III strains

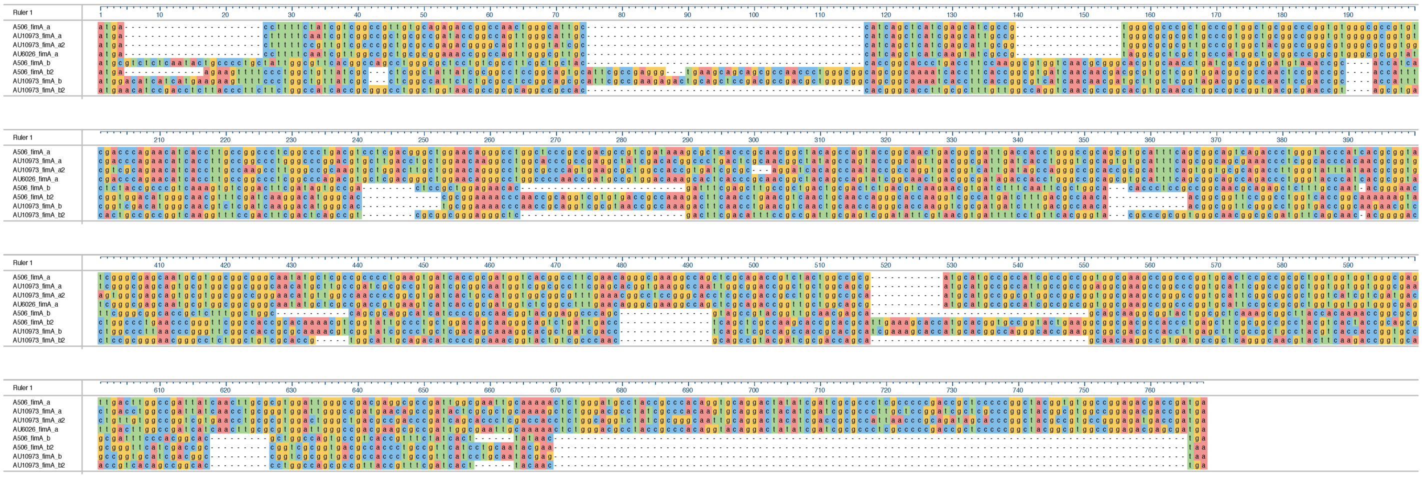

Supplement: Additional file 8: Figure S2. — Alignment of fimA homologues from select environmental and clinical subclade III strains. fimA homologues defined in RAST [78]. Nucleotide sequence of fimA homologues aligned with the MAFFT algorithm within DNAstar’s MegAlign Pro software. A506 is a representative environmental strain; AU6026 and AU10973 are representative clinical strains. (PDF 1267 kb) [file 12864_2015_2261_MOESM8_ESM.pdf]
