# Supplementary material for: Comparative genomics of Pseudomonas fluorescens subclade III strains from human lungs
Source: BMC Genomics. 2015 Dec 7;16:1032. doi: 10.1186/s12864-015-2261-2 (PMC4672498; doi:10.1186/s12864-015-2261-2)

Additional File 9. Phylogenetic tree inferred from the nucleotide acid sequences of *vasH* homologues from representative environmental and clinical subclade III strains.

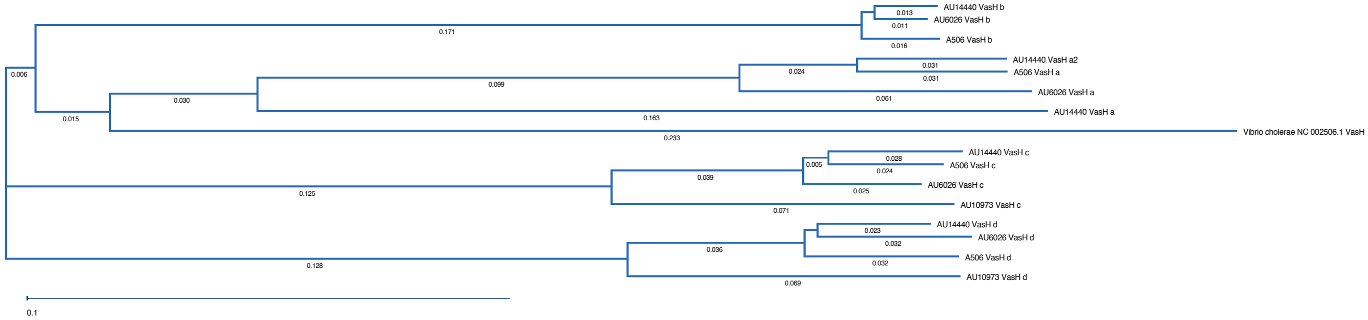

Supplement: Additional file 9: Figure S3. — Phylogenetic tree inferred from the nucleotide acid sequences of vasH homologues from representative environmental and clinical subclade III strains. Mauve algorithm used for alignment [88]. A506 is a representative clinical strain; AU6026, AU10973, AU14440 are representative clinical strains. (PDF 346 kb) [file 12864_2015_2261_MOESM9_ESM.pdf]
